# A New Analytical Method for Determination of Cathepsin L Based on the Surface Plasmon Resonance Imaging Biosensor

**DOI:** 10.3390/ijms20092166

**Published:** 2019-05-01

**Authors:** Anna Tokarzewicz, Lech Romanowicz, Anna Sankiewicz, Adam Hermanowicz, Krzysztof Sobolewski, Ewa Gorodkiewicz

**Affiliations:** 1 Department of Medical Biochemistry, Medical University of Bialystok, A. Mickiewicza 2C, 15-089 Bialystok, Poland; lech.romanowicz@umb.edu.pl (L.R.); zdbioch@umb.edu.pl (K.S.); 2 Department of Electrochemistry, Institute of Chemistry, University of Bialystok, Ciolkowskiego 1K, 15-245 Bialystok, Poland; ania@uwb.edu.pl (A.S.); ewka@uwb.edu.pl (E.G.); 3 Department of Pediatric Surgery and Urology, Medical University of Bialystok, Waszyngtona 17, 15-274 Bialystok, Poland; ahermanowicz@wp.pl

**Keywords:** cathepsin L, Surface Plasmon Resonance Imaging technique, biosensors, endopeptidase

## Abstract

The purpose of this study was to develop a new method for a determination of the cathepsin L—biosensor based on the Surface Plasmon Resonance Imaging technique. The cathepsin L is an endopeptidase, which degrades proteins and plays an important role in various processes occurring in the human body. The detection technique, Surface Plasmon Resonance Imaging, is an optical, label-free technique, which can be used for quantitative determination of the different proteins. In order to bind the enzyme, the cathepsin L inhibitor—RKLLW-NH_2_ was used. The validation process showed that parameters: precision, accuracy, and selectivity of the method were acceptable. The analytically useful range of the standard curve was 0.50 ng/mL—15.00 ng/mL. The detection and quantification limit of method was 1.67 pg/mL and 5.07 pg/mL, respectively. The usefulness of the developed method was confirmed by the determination of the cathepsin L concentration in the blood plasma of some healthy persons and in the blood plasma of patients. The obtained results were compared with the results obtained by the ELISA. It was found that the correlation between these two methods was very strong, what suggest that the developed method can be used as the competitive method to the ELISA.

## 1. Introduction

Cathepsin L (CTSL, EC 3.4.22.15) is classified as papain-like cysteine endopeptidase. The family of these enzymes consists of 55 representatives, of which the main ones are: cathepsin L, cathepsin B, F, H, K, O, S, T, V, as well as chymopapain, papain, ficin, caspase numbered from 1 to 11, and calpains numbered from 1 to 3 [1,2].

Lysosomal cathepsin L, B, and H are the best-known compounds of this family. These enzymes hydrolyze peptide bonds with the active residue of a cysteine in their active center. They exhibit the maximum of catalytic activity in the acid environment [3,4].

Cysteine proteases are produced in the cells of bones, blood vessels, muscles, and also by macrophages and others. They are involved not only in a physiological process of protein degradation, prohormone processing, immune responses, and extracellular matrix remodeling of a bone [2], but also in carcinogenesis processes at many levels: they participate in neoplastic transformation, invasion, and metastasis. The possibility of using the activity measurements of these enzymes as markers of “tumor aggressiveness” and measuring the activity of its inhibitors as markers of “defense of the body” in cancer diagnostics and therapy monitoring was confirmed. In order to maintain the physiological balance of the body, it is necessary to maintain a balance between the activity of proteases and the activity of protease inhibitors—cystatins. The disruption of this balance leads to the development of many physiological abnormalities, including the development of cancer [5].

Cathepsin L is one of the most active cysteine proteases. It degrades: collagen, elastin, the alpha-1 protease inhibitor, which controls neutrophil elastase—enzyme which destroys bacteria—and is produced during the inflammatory process [6,7].

This protease is involved in physiological processes, such as endosomal protein degradation or antigen presentation. It is also an important regulator and signal molecule in many biological processes, for example, in the formation of thyroid hormones [8,9].

Cathepsin L (CTSL) is involved in the development of diseases such as myofibril necrosis in myopathy and ischemic myocardial disease, rheumatoid arthritis [10,11], renal tubular reactions in the course of proteinuria [12] and in osteoporosis [6]. It was also found that CTSL is involved in neoplastic transformation as well as invasion and metastasis [5].

As a detection method for determination of the CTSL concentration in biological samples, Surface Plasmon Resonance Imaging (SPRI) technique was used. It can be concluded that this technique can be an alternative method for estimation of the CTSL to same quantitative and semi-quantitative methods, e.g., immunohistochemistry (ELISA) [13], spectrophotometry [3], or western blot [14].

SPRI is a label-free, optic technique, which is based on the dependence of light reflectivity changes during the adsorption of the molecules on the metal surface, if the conditions of surface plasmon resonance are fulfilled. This method is based on a resonant oscillation of conduction electrons (surface plasmon polaritons—non-radiative electromagnetic plasmon wave) at the interface between negative and positive permittivity material stimulated by incident light (laser light). Each binding to the biosensor surface biological particles causes changes in the plasmon wave. In the picture of the biosensor this process can be observed as a change in the intensity of light reaching the charge-coupe device (CCD) camera [15,16]. From the previously prepared standard curve the concentrations of the CTSL can be determined.

During the quantitative determination of the different proteins by the SPRI technique two, different types of an immobilization are used: antibody—antigen (covalent type of the immobilization, e.g., [17]) or inhibitor—enzyme (hydrophobic type of the immobilization, e.g., [18]). For the quantification of the CTSL, the enzyme—inhibitor (RKLLW-NH_2_) interaction was used.

The RKLLW-NH_2_ (C_35_H_59_N_11_O_5_) is a synthetic, commercially available inhibitor, which is composed of the following amino acids: Arg-Lys-Leu-Leu-Trp-NH_2_ (pentapeptide). This compound has cyclic rings and hydrophobic hydrocarbon chains fragments that have been used to generate hydrophobic interactions with 1-octadecanothiol (ODM). This made a possibility to immobilize this compound on the surface of the biosensor and use it as a receptor necessary for binding CTSL—hydrophobic immobilization. It has been shown that this pentapeptide inhibits the human cathepsin L even at nanomolar concentrations [19].

The aim of this study was to develop and to validate the SPRI biosensor for the determination of CTSL concentration in biological samples.

## 2. Results

### 2.1. Optimization of RKLLW-NH_2_ Concentration

In order to bind the CTSL from the sample, it was necessary to immobilize a suitable biological receptor layer—the selective CTSL inhibitor—RKLLW-NH_2_. Due to the structure of the inhibitor—hydrophobic fragments – it was decided to use the hydrophobic type of immobilization. This process consisted of two steps. The first one was formation of the linker monolayer, which was ODM (1-octadecanothiol). It was done by immersion of the chip for at least 24 h at a room temperature in 20 mM ODM ethanolic solution. Next, the chip was rinsed with ethanol and water and dried with a stream of an argon. The second step was creating of the hydrophobic bonds between RKLLW-NH_2_ and ODM, which gave the possibility to bind the inhibitor on the surface of the biosensor.

To optimize the concentration of the CTSL inhibitor on the biosensor surface—optimal saturation, two µL of nine different RKLLW-NH_2_ water solutions (1.0–100.0 ng/mL) were placed on each of the nine active places modified by ODM and incubated for 24 h at 37 °C. After this time, the surface of the biosensor was washed twice with HBS-ES buffer and then at least ten times with distilled water.

Next, the CTSL standard solution 10.00 ng/mL in acetate buffer at pH = 4.5 (CTSL shows the maximum activity at the acidic pH, which was provided by this buffer [3]), was placed onto the biosensor surface for 10 min interaction at the room temperature. After this time the biosensor was rinsed with HBS-ES buffer and water (at least 10 times) and dried in the air. The SPRI measurement was performed immediately after the preparation of the biosensor.

**Figure 1:**
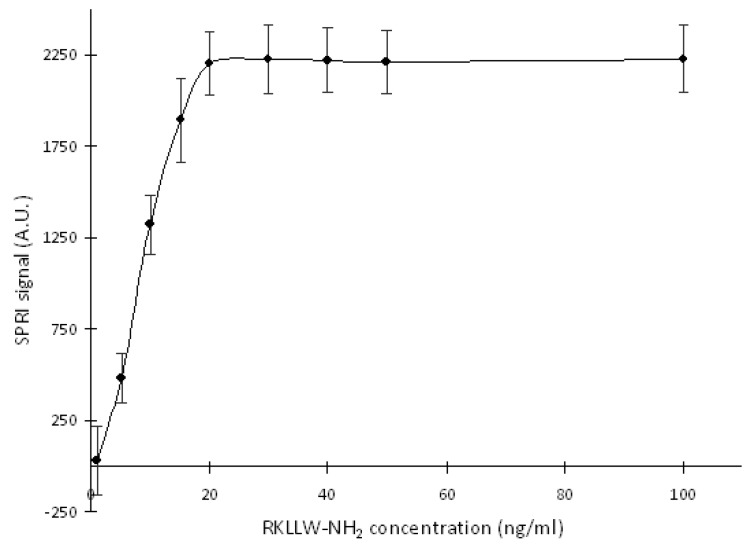
The dependence of the Surface Plasmon Resonance Imaging (SPRI) signal (A.U.) on RKLLW-NH_2_ concentration. Cathepsin L (CTSL) standard solution concentration is 10.00 ng/mL; pH = 4.5. Error bars are the standard deviation of the average SPRI signal of each standard solution determined from twelve independent measurements.

Figure 1 shows obtained results of the biosensor surface saturation by the inhibitor. Based on the obtained curve, of which the plateau part was observed for the RKLLW-NH_2_ concentration above 20.00 ng/mL, the concentration 30.00 ng/mL of the inhibitor was selected as the optimal for quantification of the enzyme and was used in further measurements.

### 2.2. Cathepsin L pH Solution Optimization

Due to the fact that cathepsin L is active in a wide pH range: 3.0–6.5 [20], it was necessary to determine the pH of the CTSL samples at which this enzyme would show maximum activity and would most effectively bind to the receptor on the surface of the biosensor.

For this purpose, eight different CTSL solutions (10 ng/mL) in buffers at pH: 2.20; 3.00; 4.00; 4.50; 4.99; 5.57; 6.52; 7.40 were prepared. Next, on the chip with the prepared monolayers of the ODM and RKLLW-NH_2_ (30.00 ng/mL) the standard CTSL solutions (10 ng/mL) at different pH with 10 min interaction time were applied. The response of the analytical SPRI signal for each of the CTSL solution was measured in twelve repetitions.

**Figure 2:**
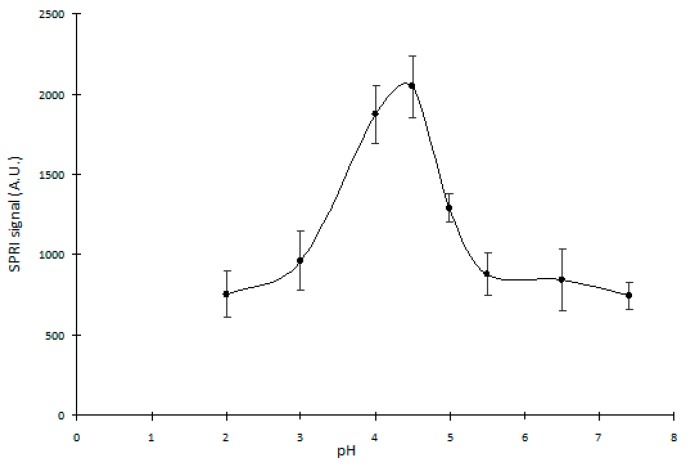
The dependence of the Surface Plasmon Resonance Imaging (SPRI) signal (A.U.) on the pH of the Cathepsin L (CTSL) solution. CTSL standard solution concentration is 10.00 ng/mL. Error bars are the standard deviation of the average SPRI signal of each standard solution determined from twelve independent measurements.

Based on the obtained data, the curve of the SPRI signal dependence on the pH of the CTSL solutions was created and is shown in the Figure 2.

Based on the results, it could be concluded that cathepsin L had a maximum activity and most effectively bound with the receptor—RKLLW-NH_2_ at pH = 4.5. For this reason, the measurements of the CTSL concentration were carried out at pH = 4.5.

### 2.3. Preparation of the Standard Curve for the Cathepsin L (CTSL) Concentration Measurments.

For the determinations of the CTSL concentration in the biological samples, preparation of a standard curve was necessary. The standard curve was the dependence of the SPRI signal (A.U.) on CTSL concentration.

**Figure 3:**
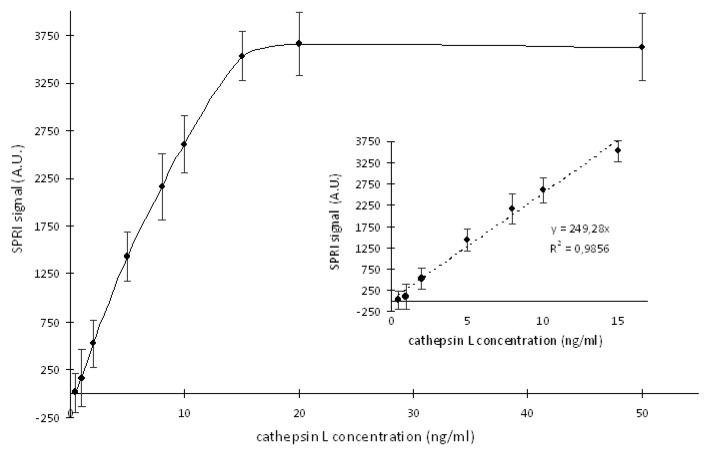
The dependence of the Surface Plasmon Resonance Imaging (SPRI) signal (A.U.) on the Cathepsin L (CTSL) concentration; the RKLLW-NH_2_ concentration was 30.00 ng/mL; pH value of solutions was 4.5; error bars are the standard deviation of the average SPRI signal of each standard CTSL solution determined from twelve independent measurements.

For this purpose, on the chip with the monolayers of the ODM and the RKLLW-NH_2_ (30.00 ng/mL) the standard CTSL solutions in concentration range of 0.5–50.0 ng/mL, at pH = 4.5, with 10 min interaction time were applied. The response of the analytical SPRI signal for each of the CTSL standard solution was measured in twelve repetitions. The obtained standard curve is shown in Figure 3.

Above the CTSL concentration of 15.00 ng/mL, the saturating of the biosensor surface was observed. Therefore, only a linear response range of the curve, between 0.50 and 15.00 ng/mL, was used for analytical purposes of the quantification of the CTSL in the samples.

The detection limit (LOD), calculated as “3× S.D./a” of the blank sample (acetate buffer, pH = 4.5), where “a” was the directional coefficient of the curve, was 1.67 pg of CTSL/mL. The limit of quantification (LOQ = 10× S.D./a) equaled to 5.07 pg of CTSL/mL.

The standard curve of the conducted in this study ELISA (enzyme-linked immunosorbent assay), was characterized by the range of 62.50–4000.00 pg/mL (manufacturer data).

### 2.4. The Selectivity of the Surface Plasmon Resonance Imaging (SPRI) Biosensor

To check whether the newly created method during the quantification measurements of the CTSL was selective, i.e., did not bind any other protein contained in the biological samples, a mixture of the CTSL with different, potential interferents were applied on the biosensor. As interferents, human albumin, MMP-1, MMP-2, cathepsin B, cathepsin D, cathepsin E, and cathepsin G were used. The choice of these interferents was made because of the fact that albumin is the most common protein in human body fluids [21]; MMP-1, MMP-2, as the CTSL, which are proteases, take part in the degradation of the extracellular proteins [17]; cathepsin B, D, E, G are compounds belonging to the cathepsin group as the cathepsin L.

The different mixtures of the CTSL (constant concentration 8.00 ng/mL) with various excesses of the human albumin, MMP-1, MMP-2, cathepsin B, cathepsin D, cathepsin E, and cathepsin G within a range 1:1 to 1:10,000 (concentration ratio) were examined. The results obtained from this part of the biosensor development are shown in Table 1.

Based on the Cathepsin-L (CTSL) concentration results, no significant influence of the human albumin, metalloproteinase-1 (MMP-1), metalloproteinase-2 (MMP-2), cathepsin B, cathepsin D, cathepsin E, and cathepsin G, even at 10,000-fold excess of the interferents, was observed. Therefore, it could be concluded that the biosensor was suitable for the CTSL concentration determination in different samples.

### 2.5. Precision and Accuracy of the Surface Plasmon Resonance Imaging (SPRI) Method

The next stage in the development of the biosensor was determination of a precision and an accuracy. The precision was tested for three different CTSL concentrations: 0.50; 5.00; 15.00 ng/mL, with 36 repetitions for each sample. The solutions pH was 4.5, the RKLLW-NH_2_ concentration was 30.0 ng/mL. The obtained results are shown in Table 2.

Based on the obtained results, it could be concluded, that the precision of mean value, as well as confidence limits were acceptable. 36 repetitions of the CTSL concentration measurement for each of the sample compensated the effect of relatively poor precision of a single measurement.

### 2.6. Cathepsin L Concentration Determination in the Biological Samples and Correlation with ELISA

In order to verify suitability of the newly created method for the CTSL quantification in biological samples, two types of the samples by the SPRI biosensor and ELISA were analyzed: nine control blood plasma samples and nine blood plasma samples of the patients collected before and after the surgical resection: ovarian tumor, ovarian cyst or gall bladder removal.

After equilibration to the room temperature, the blood plasma samples were applied onto the biosensor to interact with the RKLLW-NH_2_ for 10 min. After the interaction, the surface of the biosensor was washed twice with HBS-ES buffer and ten times with distilled water.

The CTSL concentration was evaluated on the basis of the linear range of the standard curve (see Figure 5). The results of the CTSL concentration measurements obtained by the SPRI biosensor as well as the ELISA were compared and are shown in Table 3.

The average results of CTSL concentration in the two types of the blood plasma obtained by ELISA and SPRI methods were similar. The results of CTSL concentration in the control blood plasma samples obtained by ELISA varied between 9.96–42.10 ng/mL, whereas the results obtained by the SPRI technique varied between 8.12–39.83 ng/mL. The results of CTSL concentration in the patients’ blood plasma varied between 13.30–26.40 ng/mL for the ELISA, and between 14.13–29.76 ng/mL for the SPRI biosensor.

In order to show the difference between results obtained by the two methods, the graphs of the linear correlation of the CTSL concentration in the biological samples were developed. Also, the Pearson product-moment correlation coefficients were calculated.

**Figure 4:**
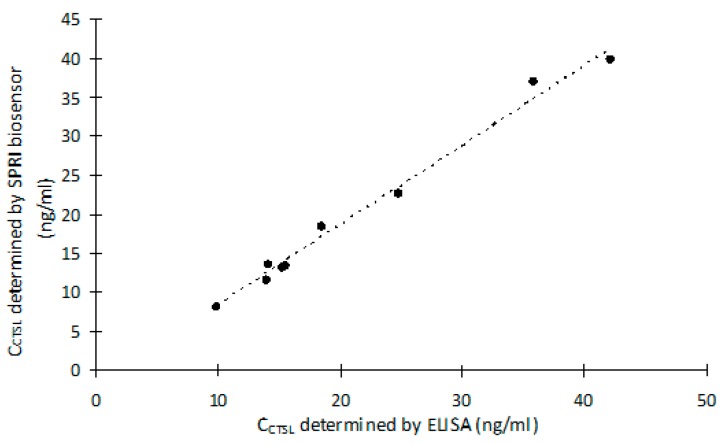
The linear correlation between the results of Cathepsin-L (CTSL) concentration in the control blood plasma samples obtained by Surface Plasmon Resonance Imaging (SPRI) method and ELISA.

Figure 4 shows the linear correlation between the results of CTSL concentration in the control blood plasma samples, obtained by the SPRI biosensor and ELISA. The Pearson product-moment correlation coefficient calculated for this correlation was 0.99.

**Figure 5:**
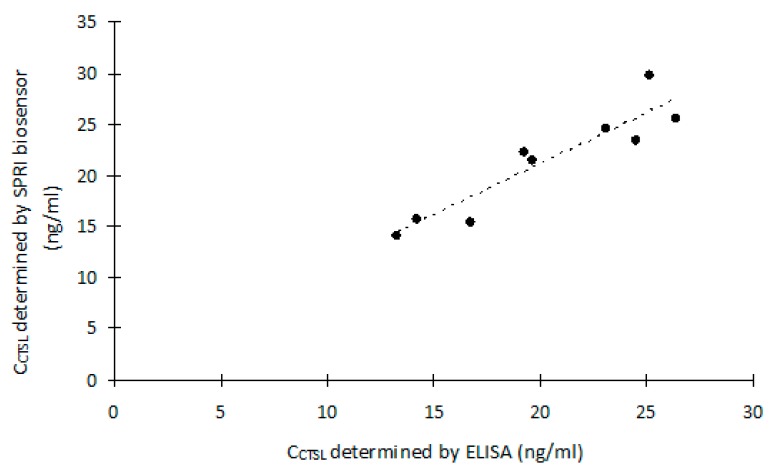
The linear correlation between the results of Cathepsin-L (CTSL) concentration in the blood plasma of the patients, obtained by Surface Plasmon Resonance Imaging (SPRI) method and ELISA.

Figure 5 shows the linear correlation between the results of CTSL concentration in the blood plasma of the patients obtained by the SPRI biosensor and ELISA. The Pearson product-moment correlation coefficient calculated for this correlation was 0.92.

## 3. Discussion 

During the study, the biosensor based on the Surface Plasmon Resonance Imaging technique was developed. For this purpose, the CTSL inhibitor (RKLLW-NH2) as a biological receptor, which bound to the chip surface via ODM linker, was used. Also, its concentration on the biosensor surface was optimized.

The validation process proved that the developed SPRI biosensor is characterized by suitable precision and accuracy, and selectivity against excess of the different interferents, e.g., cathepsin D. Also, the linear range of the standard curve, which was necessary for the CTSL quantitative determination, was determined.

To confirm the utility of the created method, the biosensor was used for the determination of the CTSL concentration in the biological samples. As a reference method, the ELISA was used. The results of the CTSL concentration measurements in the two different types of the blood plasma samples obtained by the developed SPRI biosensor are closely similar to those obtained by the ELISA. The Pearson product–moment correlation coefficients, determined for these two methods in both types of the samples, were very close to 1, which showed almost total positive linear correlations. It should be taken into account that the individual characteristic of the patients could have influence on the obtained results.

Generally, the ELISA is characterized by high sensitivity and specificity. Therefore, it is the most common method for quantitative determination of a different compound in biological samples. However, during the measurements, several advantages of the SPRI over the ELISA were noted.

The ELISA was time consuming. The total measurement time using this method was about six hours. Also, ELISA consumed large quantities of the samples and the reagents, e.g., 100 mL of each sample and about 1 l of the washing buffer were used. In order to maintain high precision and accuracy, it was necessary to repeat measurement of each same sample several times. Therefore, it was concluded that ELISA is quite expensive per one sample. Moreover, it should be stressed that it used light sensitive chemical labels, which could cause artefacts during the measurements.

The SPRI biosensor overcomes some of these drawbacks. The chips with immobilized ODM monolayer could be stored for at least two weeks without changes in the ODM properties, which significantly shortened the measurement time. Such a method as the ELISA could detect very low quantities of the reagents with high specificity. SPRI measurements were not based on the labels or the addition of the second reagent and enabled the measurement of nine samples simultaneously in real time. Total SPRI biosensor measurement time was about one and a half hours per chip. Also, it should be stressed that for each of the sample 12 independent repetitions were done, which significantly increased the precision and accuracy of the measurement. Moreover, the SPRI technique used smaller quantities of the samples and reagents, e.g., 2.5 mL of each sample, 100 mL of the distilled water. Therefore, it could be concluded that the SPRI biosensor method can be competitive to the ELISA.

The different surface modifications and bioanalytical procedures are used for ELISA and SPRI, which may limit the actual analytical comparison of these techniques.

## 4. Materials and Methods

### 4.1. Materials

As a standard: cathepsin L from the human liver (M = 24.2 kDa), RKLLW-NH_2—_selective cathepsin L inhibitor (Arg-Lys-Leu-Leu-Trp-NH_2_, M = 405.50 kDa), recombinant matrix metalloproteinase-2 (MMP-2), human albumin, cathepsin D, cathepsin G, 1-octadecanothiol (ODM, all Sigma Steinheim, Germany), recombinant matrix metalloproteinase-1 (MMP-1, Wuhan USCN, Hubei), cathepisn B, cathepsin E, (all Calbiochem, Merck Ltd.) were used. Photopolymer ELPEMER SD 2054, hydrophobic protective paint SD 2368 UV SG-DG (PETERS, Kempen, Germany) were used. Also, HBS-ES buffer pH = 7.4 (0.01 M HEPES, 0.15 M sodium chloride, 0.005% Tween 20, 3 mM EDTA), phthalate buffer pH: 2.20, 3.00, acetate buffer pH: 4.0, 4.5, 4.99, 5.57, Michaelis phosphate buffer pH: 6.52, 7.40 (all BIOMED, Lublin, Poland) were used. The aqueous solutions were prepared with miliQ water (Simplicity^®^MILLIPORE). Absolute ethanol, acetic acid, sodium chloride, sodium acetate (all POCh, Gliwice, Poland), high purity (99.999%) argon N 5.0 (AIR LIQUIDE, Poland), and ELISA kit (cat. No SEA306Hu, Cloud—Clone Corp., USA) were used.

### 4.2. Types and Preparation of the Biological Samples

All blood samples of the patients were collected before and after the surgical resection: ovarian tumor, ovarian cyst, or gall bladder removal during the hospital admission to the L. Zamenhof Children’s Clinical Hospital of the Medical University of Bialystok, Pediatric Surgery Department (Bialystok, Poland). Ethylenediaminetetraacetic acid (EDTA) was used as an anticoagulant. The blood samples of the control group were taken from the honorary blood donors of the Regional Blood Donor Centre of Bialystok, who were confirmed to be healthy.

Two mL of each blood sample was centrifuged (1000× *g*) for over 15 min, filtered three times for the separation of plasma from the blood cells, frozen, and stored at −70 °C until further use.

All subjects gave their informed consent for inclusion before they participated in the study. The study was conducted in accordance with the Declaration of Helsinki, and the protocol was approved by the Ethics Committee of Medical University of Bialystok, project identification code: R-I-002/63/2013 (16 June 2013).

### 4.3. Chip Preparation for the Measurements

**Figure 6:**
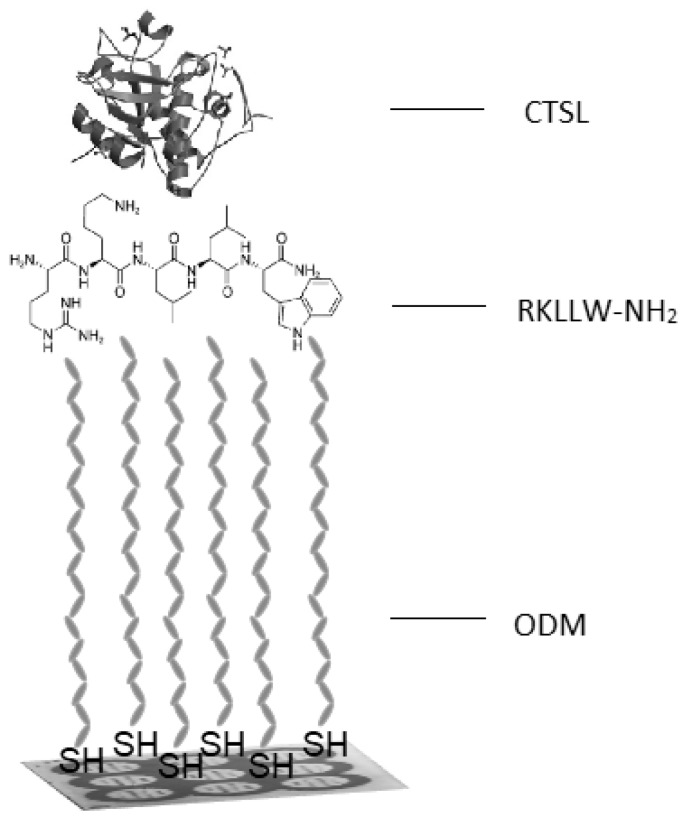
The schematic illustration of the biosensor for the quantitative measurements of the CTSL. ODM—1-octadecanothiol, RKLLW-NH_2_—CTSL inhibitor, CTSL—cathepsin L.

The gold chips were manufactured, and its gold surface was covered by photopolymer and hydrophobic paint as described in the previous paper [18,22,23,24]. Each chip prepared in this way had nine active places and each place had twelve measurement spots. It gave the possibility to measure nine different samples (solutions) simultaneously in twelve repetitions of the SPRI signal for each of the sample without intermixing them. A schematic picture of the biosensor for the measurements of the CTSL concentration is presented in Figure 6.

### 4.4. Scanning Electron Microscopy (SEM) Measurements

To control and confirm the formation of subsequent layers, i.e., gold (as prepared), ODM, RKLLW-NH_2_ and immobilized CTSL on the surface of the biosensor, the Scanning Electron Microscope (SEM) measurements were performed.

For this purpose, a commercial SEM Phenom ProX was used. Parameters and equipment of the SEM used for the biosensor layers examination are as follows: CeB_6_ as an electron source, backscattered electron detector, accelerating voltage 10 kV, magnifications 5000×.

**Figure 7:**
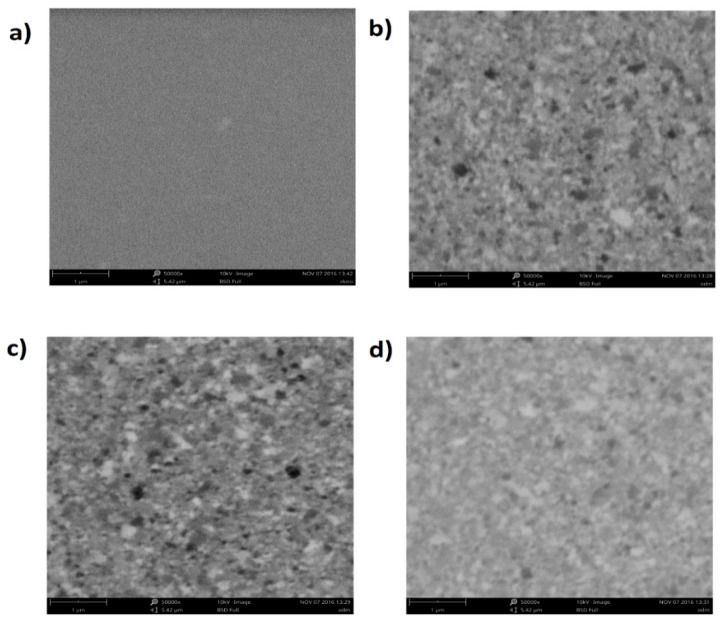
Scanning electron microscopy (SEM) images of the individual layers on the gold chip surface, (**a**) the bare gold; (**b**) 1-octadecanothiol (ODM); (**c**) cathepsin L inhibitor (RKLLW-NH_2_). (**d**) Cathepsin L (CTSL).

Based on the obtained SEM pictures presented in Figure 7, the creation of the different structures after each stage could be observed. Therefore, it may be concluded that the formation of each layer: bare gold—Figure 7a, ODM—Figure 7b, RKLLW-NH_2_—Figure 7c and immobilized CTSL—Figure 7d, took place on the biosensor surface.

### 4.5. Basis of the Cathepsin L Quantification and Optimization of Surface Plasmon Resonance Imaging (SPRI) Measurement

The schematic illustration of the SPRI apparatus and the principles of measurements, where it had been used, were presented in the previous paper [25].

To determine CTSL concentration, it was necessary to measure interaction between the CTSL and the inhibitor at a proper angle, i.e., where the contrast between the background and the active sites was the largest. For this purpose, two images of the biosensor surface by a CCD (charge-coupled device) camera were taken. The first one was a photo with the immobilized RKLLW-NH_2_ layer on it. The other one was the biosensor surface photo taken after the interaction of the CTSL solution with the inhibitor.

The SPRI signal was calculated by comparing the two intensities of the reflected light from the two biosensor layers: RKLLW-NH_2_ and immobilized CTSL, for each spot separately. The contrast values obtained for all pixels across a single spot of the particular sample were integrated. Then, the SPRI signal was integrated over the whole spot area. The digital image processing software ImageJ [26] version 1.32 was used to evaluate the SPRI images in 2D form and to digitize the signal intensity (in A.U.).

To monitor non-specific binding during the measurements, three different procedures were used: measuring the SPRI signal in a place on the chip without the receptor, preparing samples in the buffer pH = 4.5 (pH near the isoelectric point of the protein), and applying buffer pH = 4.5 on the some of the places on the biosensor.

In the analytically useful range of the standard curve (linear part of the standard curve), the SPRI signal was proportional to the CTSL amount bound with the immobilized receptor’s layer on the biosensor surface. This dependence gave possibility to determine concentration of the CTSL in the samples.

## 5. Conclusions

In general, the advantages of the SPRI biosensor over ELISA were found. Also, a good correlation between results obtained by these two methods was determined. Therefore, CTSL SPRI biosensor could be used in laboratories for a serial quantitative determination.

## Figures and Tables

**Table 1:** Influence of human albumin, metalloproteinase-1 (MMP-1), metalloproteinase-2 (MMP-2), cathepsin B, cathepsin D, cathepsin E, and cathepsin G as interferents on the CTSL quantification measurements. Concentration of the Cathepsin-L (CTSL) standard was 8.00 ng/mL (*n* = 12).

Interferent	C_CTSL_: C_interferent_	Measured C_CTSL_ ± S.D. [ng/mL]	Recovery [%]
Albumin	1:1000	7.89 ± 0.69	99
	1:10,000	8.12 ± 0.39	101
MMP-1	1:1	8.15 ± 0.47	102
	1:10	8.50 ± 0.55	106
MMP-2	1:100	7.93 ± 0.40	99
Cathepsin B	1:1	8.13 ± 0.08	102
	1:10	7.95 ± 0.26	99
Cathepsin D	1:1	8.37 ± 0.20	105
	1:10	8.05 ± 0.17	101
Cathepsin E	1:1	8.06 ± 0.40	101
	1:10	8.32 ± 0.25	104
Cathepsin G	1:1	7.92 ± 0.28	99
	1:10	8.03 ± 0.36	100

**Table 2:** Precision and accuracy of the Cathepsin-L (CTSL) quantification by Surface Plasmon Resonance Imaging (SPRI) method.

Number of Measuring Points	Tested C_CTSL_ [ng/mL]	Measured C_CTSL_± S.D. [ng/mL]	Recovery [%]	Confidence Limit (95%) [ng/mL]
36	0.50	0.51 ± 0.15	102	0.12
36	5.00	5.07 ± 0.23	101	0.19
36	15.00	15.48 ± 0.55	103	0.44

**Table 3:** The Cathepsin-L (CTSL) concentration in the different types of the human blood plasma determined by the Surface Plasmon Resonance Imaging (SPRI) biosensor and ELISA.

Number.	C_CTSL_ in Control Blood Plasma (ng/mL)		C_CTSL_ in Blood Plasma of Patients (ng/mL)	
	ELISA	SPRI	ELISA	SPRI
**1**	24.84 ± 0.25	22.55 ± 0.28	13.30 ± 0.13	14.13 ± 0.31
**2**	42.10 ± 0.42	39.83 ± 0.54	25.12 ± 0.25	29.76 ± 0.65
**3**	14.16 ± 0.14	13.45 ± 0.14	16.74 ± 0.17	15.36 ± 0.72
**4**	9.96 ± 0.10	8.12 ± 0.56	14.18 ± 0.14	15.74 ± 0.26
**5**	18.48 ± 0.18	18.43 ± 0.61	24.54 ± 0.25	23.38 ± 0.58
**6**	35.84 ± 0.36	36.84 ± 0.29	26.40 ± 0.26	25.43 ± 0.52
**7**	15.56 ± 0.16	13.25 ± 0.29	23.08 ± 0.23	24.53 ± 0.47
**8**	15.26 ± 0.15	13.14 ± 0.73	19.62 ± 0.20	21.41 ± 0.86
**9**	13.98 ± 0.14	11.58 ± 0.48	19.22 ± 0.19	22.27 ± 0.45
Average	21.13 ± 10.99	19.69 ± 11.36	20.24 ± 4.83	21.34 ± 5.27
Range	9.96–42.10	8.12–39.83	13.30–26.40	14.13–29.76
